# Adipose tissue as target of environmental toxicants: focus on mitochondrial dysfunction and oxidative inflammation in metabolic dysfunction-associated steatotic liver disease

**DOI:** 10.1007/s11010-024-05165-z

**Published:** 2024-12-20

**Authors:** Bogdan M. Lolescu, Adina V. Furdui-Lința, Cosmin A. Ilie, Adrian Sturza, Flavia Zară, Danina M. Muntean, Alexandru Blidișel, Octavian M. Crețu

**Affiliations:** 1 Doctoral School Medicine, Center for Translational Research and Systems Medicine, “Victor Babeș” University of Medicine and Pharmacy of Timișoara, Timișoara, Romania; 2 Center for Translational Research and Systems Medicine, “Victor Babeș” University of Medicine and Pharmacy of Timișoara, Timișoara, Romania; 3 Department III Functional Sciences–Chair of Pathophysiology, “Victor Babeș” University of Medicine and Pharmacy of Timișoara, Timișoara, Romania; 4 Department III Functional Sciences–Chair of Public Health & Sanitary Management, “Victor Babeș” University of Medicine and Pharmacy of Timișoara, Timișoara, Romania; 5 Department II Microscopic Morphology–Chair of Histology, “Victor Babeș” University of Medicine and Pharmacy of Timișoara, Timișoara, Romania; 6 Department of Pathology, Timisoara Municipal Emergency Clinical Hospital, Timișoara, Romania; 7 Department of Surgery I–Clinic of Surgical Semiotics & Thoracic Surgery, Center for Hepato-Biliary and Pancreatic Surgery, “Victor Babeș” University of Medicine and Pharmacy of Timișoara, Eftimie Murgu Sq., No.2, 300041 Timișoara, Romania

**Keywords:** Endocrine disruptors, Micro-/nano-plastics, Mitochondrial dysfunction, Oxinflammation, Adipocytes, Adipose tissue, Liver, Metabolic dysfunction-associated steatotic liver disease (MASLD)

## Abstract

Obesity, diabetes, and their cardiovascular and hepatic comorbidities are alarming public health issues of the twenty-first century, which share mitochondrial dysfunction, oxidative stress, and chronic inflammation as common pathophysiological mechanisms. An increasing body of evidence links the combined exposure to multiple environmental toxicants with the occurrence and severity of metabolic diseases. Endocrine disruptors (EDs) are ubiquitous chemicals or mixtures with persistent deleterious effects on the living organisms beyond the endocrine system impairment; in particular, those known as metabolism-disrupting chemicals (MDCs), increase the risk of the metabolic pathologies in adult organism or its progeny. Being largely lipophilic, MDCs mainly target the adipose tissue and elicit mitochondrial dysfunction by interfering with mitochondrial bioenergetics, biogenesis, dynamics and/or other functions. Plastics, when broken down into micro- and nano-plastics (MNPs), have been detected in several human tissues, including the liver. The harmful interplay between inflammatory and redox processes, which mutually interact in a positive feed-back loop, hence the term oxidative inflammation ("OxInflammation"), occurs both at systemic and organ level. In both liver and adipose tissue, oxinflammation contributes to the progression of the metabolic dysfunction-associated steatotic liver disease (MASLD). Moreover, it has been reported that individuals with MASLD may be more susceptible to the harmful effects of toxicants (mainly, those related to mitochondria) and that chronic exposure to EDs/MDCs or MNPs may play a role in the development of the disease. While liver has been systematically investigated as major target organ for ambient chemicals, surprisingly, less information is available in the literature with respect to the adipose tissue. In this narrative review, we delve into the current literature on the most studied environmental toxicants (bisphenols, polychlorinated biphenyls, phthalates, tolylfluanid and tributyltin, per-fluoroalkyl and polyfluoroalkyl substances, heavy metals and MNPs), summarize their deleterious effects on adipose tissue, and address the role of dysregulated mitochondria and oxinflammation, particularly in the setting of MASLD.

## Introduction

Endocrine disruptors (EDs) are exogenous compounds or mixtures, mostly chemicals but also plant hormones/toxicants, classically reported to interfere with the normal function of the endocrine system due to their chronic environmental presence. EDs are very heterogeneous and can be found in everyday products, from consumer products (e.g., cans, plastics, cosmetics, etc.) to electronics and agricultural pesticides.

In the past decades, elucidation of the pathophysiological mechanisms underlying the deleterious effects of EDs has become a growing field of research, since they have been associated with a diverse array of chronic health issues. The rates of obesity, metabolic syndrome, type 2 diabetes, and the related liver comorbidities have exponentially risen worldwide over the past decades, generating substantial societal costs [1]. The environmental toxicants have been increasingly implicated in the global decline in metabolic health besides the classic risk factors for these pathologies [2, 3]. In particular, those known as metabolism-disrupting chemicals (MDCs) or obesogens have been systematically associated in the past decade with an increased risk of metabolic diseases in adult organism or its progeny (comprehensively reviewed in refs. [4, 5]).

Plastics play a significant role in various aspects of daily life, including technology, medicine, treatments, and domestic appliances. The vast quantities of plastics discarded daily break down into micro- and nano-sized particles, raising concerns about their toxicity to the environment and human health [6–8]. Microplastics (MPs) are typically defined as plastic particles between 1 and 5000 µm in size, while nano-plastics (NPs) are considered to have a size between 1 and 1 µm [9]. Over the past few decades, a great deal of research has been aimed at elucidating the toxic effects of micro- and nano-plastics (MNPs) in plants, animals, and humans [10]. As such, MNPs have been shown to have detrimental effects on several organs in both rodent experiments and human samples [11, 12]. More important, MNPs have been recently found in a variety of human tissues and organs, including blood, liver, kidney, placenta, lungs, and atheromas [13, 14].

Biodegradable plastics are gaining public attention as potential alternatives to nondegradable plastics, which have generated a significant issue of white pollution due to their long-lasting nature. A further concerning issue is that biodegradable polymers may undergo disintegration into MPs at a faster pace than conventional plastics, thus posing an additional environmental problem [12]. In addition to their own toxicity, biodegradable MPs (like their traditional counterparts) have the ability to absorb and serve as vector for a wide range of pollutants (e.g., phthalates, organotins, triclosan, and bisphenol), which will be released during their degradation and exert deleterious effects into the body after the MPs ingestion [15, 16].

Since the liver is the central organ involved in carbohydrates and lipids metabolism and is reached by xenobiotics, several reviews tackling the deleterious hepatic effects of ambient chemicals have been recently published [17–19]. Of note, in the liver, MNPs have been reported to increase oxidative stress and impair lipid metabolism [20], as reported for obesogens [21], thus increasing the risk for cumulative toxicity. However, being largely lipophilic, EDs/MDCs mainly target the adipose tissue and elicit mitochondrial toxicity by interfering with mitochondrial bioenergetics, biogenesis, dynamics, and other functions [22, 23].

Oxidative stress and inflammation or “oxinflammation” are a newly coined term describing the harmful interplay between inflammatory and redox processes that contribute to various diseases [24]. The pathophysiology of metabolic diseases is dominated by a self-sustaining vicious circle of persistent low-grade inflammation, increased oxidative stress, and impaired energy homeostasis via mitochondrial dysfunction, all three pathomechanisms leading to insulin resistance [25, 26].

This narrative review will focus on the impact of endocrine disruptors and micro-/ nano-plastics on mitochondria within the adipose tissue and also will briefly address their contribution to oxidative stress and inflammation in both adipose tissue and liver, particularly in the setting of the constantly rising metabolic dysfunction-associated steatotic liver disease (MASLD).

## Data sources

PubMed and Google Scholar literature search was conducted using a combination of “endocrine disruptors,” “environmental toxicants,” “metabolism-disrupting chemicals,” “obesogens,” “microplastics, “nanoplastics,” “mitochondria dysfunction,” “mitochondria toxicity,” “oxidative stress,” “inflammation,” “non-alcoholic fatty liver disease,” “metabolic dysfunction-associated steatotic liver disease”/ “MASLD,” and related terms. Titles and abstracts were screened and relevant full-text articles published in English to August 2024 were included.

## Oxidative stress and inflammation in the adipose tissue

Oxidative stress is defined as the imbalance between reactive oxygen species (ROS) production and elimination [27]. Acute inflammation represents a non-specific physiological response of the host to an infection or various tissue injuries, serving to eradicate the threat and restore tissue homeostasis while mitigating further damage [28]. At variance, chronic low-grade (sterile) inflammation has been associated with the onset and progression of most non-communicable diseases, in particular the cardiometabolic pathologies [29] among middle-aged and old adults [30].

Mitochondria are the key organelles with complex metabolic functions [31] that integrate and perpetuate in pathological settings the signal transduction pathways of both oxidative stress [32] and chronic inflammation leading to the inflammasome formation and secretion of pro-inflammatory cytokines [33], hence, the term “mito-inflammation” [34].

The dysfunctional adipose tissue contributes to the oxinflammation process through several pro-and anti-inflammatory mediators and signal transduction pathways that are intertwined in a complex crosstalk, particularly in the expanding visceral adipose depot [35]. Both the dysfunctional adipocytes and the infiltrating immune cells secrete inflammatory adipokines and cytokines that significantly contribute to the perpetuation of oxinflammation and also of mitochondrial and metabolic dysfunction. Adiponectin is the most studied anti-inflammatory adipokine whose levels decrease in obesity, reducing its protective effects. Leptin, the most studied deleterious adipokine, promotes local inflammation by activating macrophages and increasing the production of pro-inflammatory cytokines such as TNF-α and IL-6 from adipocytes and macrophages, and thus driving the systemic (sterile) inflammation. In the setting of obesity, the increased infiltration with monocytes, which subsequently differentiate into macrophages, and the polarization of macrophages toward the pro-inflammatory phenotype lead to persistent local inflammation and insulin resistance [36]. Nuclear factor-kappa β (NF-κβ) and mitogen-activated protein kinase (MAPK) pathways are common inflammatory signaling pathways. NF-κβ is a key transcription factor that, in response to signals like TNF, IL-1β, and ROS, activates transcription of pro-inflammatory genes and inhibits insulin signaling [37]. The MAPK pathways (ERK, JNK, p38) are activated by stress signals and cytokines, with JNK and p38 playing significant roles in the adipose tissue inflammation [38]. Toll-like receptors (TLRs), notably TLR4, detect ligands such as lipopolysaccharide (LPS) and saturated fatty acids, further activating NF-κB and MAPK pathways and exacerbating local inflammation. Obesity-related adipocyte hypertrophy and hyperplasia render the adipose tissue hypoxic and together with remodeling and fibrosis of the extracellular matrix further exacerbates the local inflammation [39].

Mitochondria are both sources and targets of ROS in mammalian cells and the related oxidative stress is responsible for mitochondrial dysfunction across various pathological conditions. They also contribute to the ROS compartmentalization and actively participate in the redox signaling network in both health and disease [40]. In an elegant study by Fazakerley et al. reported that oxidative stress is confined to mitochondria (not to cytosol) in 3T3-L1 adipocytes and is responsible for the impairment of insulin-dependent glucose transporter 4 (GLUT4) translocation to the plasma membrane and glucose uptake, respectively [41].

Also, mitochondria-generated ROS together with those derived from other enzymatic sources, in particular the NADPH oxidases, modulate inflammatory signaling pathways, underscoring their involvement in multiple facets of the inflammatory response, in line with the concept of “mito-inflammation” [34].

The unequivocal hallmark of the dysfunctional adipose tissue in the pathogenesis of obesity is that the concomitant occurrence of oxinflammation with mitochondrial dysfunction and impaired insulin signaling, all these pathomechanisms contributing to the various types of cell death [37]. Deciphering the possibility to modulate these pathomechanisms is crucial for developing therapeutic approaches aimed at mitigating oxinflammation in the setting of the widespread metabolic disorders.

## Oxidative stress and inflammation in the liver

Oxinflammation affects not only the adipose tissue, but also impacts the liver, and a bidirectional crosstalk has been reported to occur between these organs in the setting of metabolic diseases [42], including in MASLD [43]. Formerly known as non-alcoholic fatty liver disease, MASLD is characterized by the accumulation of fat in more than 5% of hepatocytes as observed on hepatic biopsy, with severity marked by fat presence exceeding 30% [44]. The etiology of MASLD is multifaceted and diverse. Along with genetic predisposition, over-nutrition and physical inactivity lead to obesity and metabolic syndrome, and MASLD has been closely linked with these pathologies. In the setting of obesity, nutrient excess and hepatic insulin resistance facilitate ectopic fat accumulation (liver steatosis), fostering low-grade inflammation (via the pro-inflammatory cytokines, TNF-α, IL-1β, IL-18, and IL-6) [45] and oxidative stress (both directly via ROS-mediated hepatocyte injury and death and indirectly, via their peroxidation products) [46]. The release of IL-1β and IL-18 is triggered via the formation/activation of the NLRP3 inflammasome, which recognizes specific signals. The caspase-1 cleavage in hepatic and adipose tissue macrophages is promoted by the NLRP3 inflammasome in response to the intracellular rise of ceramide, which is attributed to lipotoxicity [47]. Adipose tissue inflammation in metabolic pathologies, known as “meta-inflammation, ” is a chronic, sterile, low-grade inflammation, affecting the metabolic control of nutrient flow in adipose tissue, liver, muscle, and pancreas, with lipotoxicity significantly contributing to insulin resistance [48] and also, a pro-atherogenic environment that precipitate the cardiovascular disease [49]. The development of insulin resistance via insulin receptor substrate 1 (IRS-1) involves IKK-mediated serine phosphorylation, leading to the activation of the IKKβ/NF-κB signaling cascade. This activation results in insulin resistance by compromising the tyrosine phosphorylation of IRS-1 and the glucose transporter type 4 (GLUT4) activity [45]. The increased pro-oxidant signaling and/or relative antioxidant dysfunction of both hepatocytes and adipocytes will also propagate the pro-inflammatory cascades and lead to steatosis, and ultimately, fibrosis of the liver [46]. Excessive triglycerides storing in hepatocytes also activates macrophages residing in the liver tissue (the Kupffer cells), which further promote local inflammation via the secretion of pro-inflammatory cytokines. Last but not least, the pro-inflammatory and pro-oxidant milieu is responsible for the activation of the hepatic stellate cells and their differentiation into myofibroblasts that secrete components of the extracellular matrix with progression of liver fibrosis [45, 49].

Another important aspect in the development of MASLD is related to the alteration of the intermediary metabolism, namely the metabolic dysfunction triggered by insulin resistance. Sterol regulatory element-binding protein-1c (SREBP-1c) is a transcription factor that controls de novo lipogenesis, which together with the inhibition of FA oxidation are ultimately responsible for the impaired lipid metabolism and dyslipidemia [44]. The mechanistic target of rapamycin (mTOR) protein, a member of the PI3K-related kinase family, forms two multiprotein complexes: mTORC1 and mTORC2. mTORC1, through its regulatory protein, manages extracellular signals, promoting anabolism and suppressing catabolism under the influence of insulin. An inverse relationship between insulin resistance, hyperlipidemia, and mTORC1 expression has been reported [45]. The dysregulation of metabolic crosstalk between the adipose tissue and liver has been recently emphasized in pregnant women with obesity [50], an observation relevant view the transgenerational effect of EDs.

**Fig. 1:**
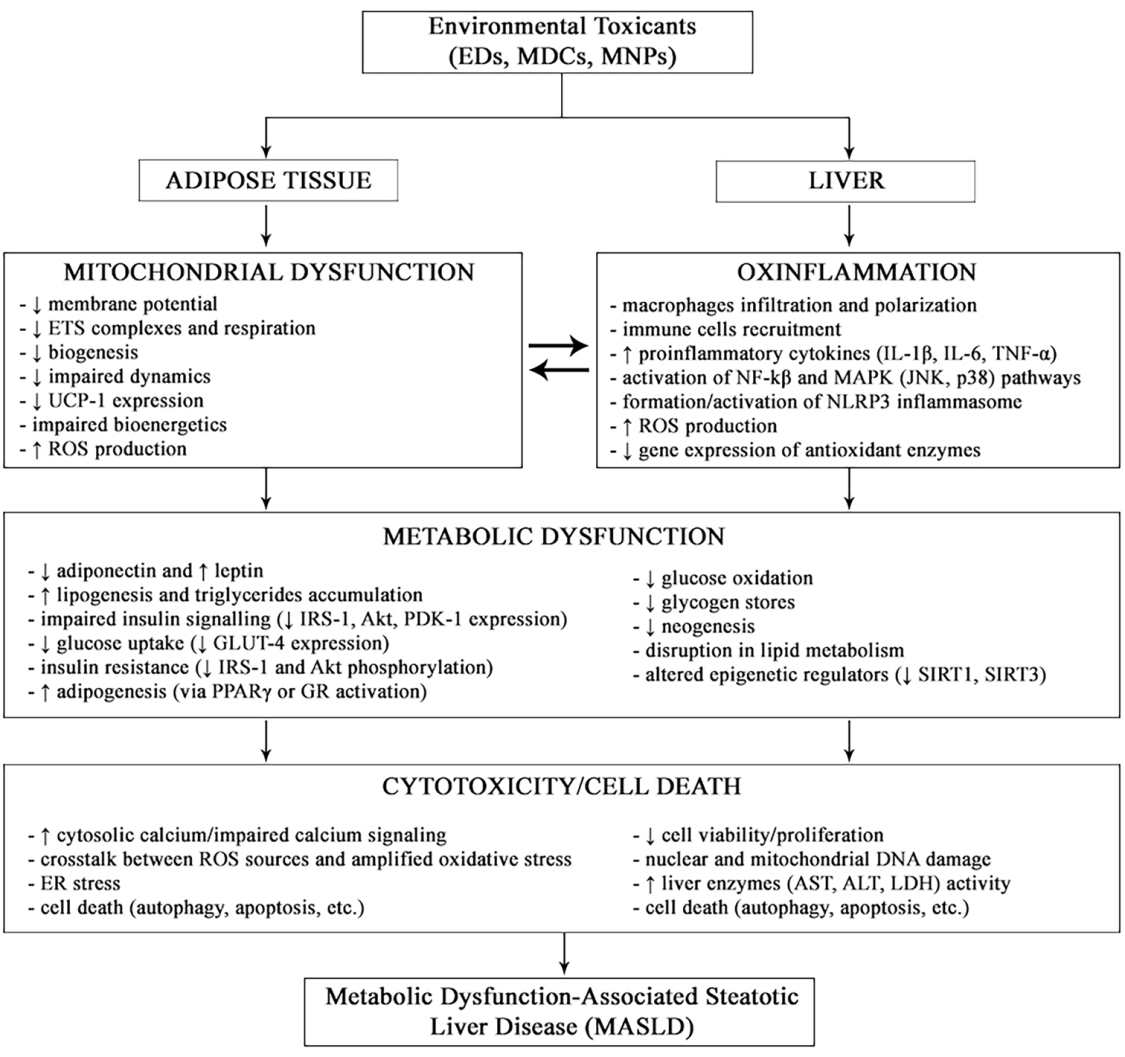
Overview of the major pathomechanisms of environmental toxicants on the adipose Tissue and liver in the setting of MASLD

An increasing body of evidence suggests that chronic environmental exposure to low levels of multiple toxicants (EDs, MDCs and MNPs) may contribute to the progression of MASLD via all the above-mentioned mechanisms (reviewed in refs. [18, 51–53]). Figure 1 provides an overview of them.

However, an increasing attention has been paid in the recent years to the mitochondrial deleterious effects of both EDs and MNPs, which are further summarized for the adipose tissue. While liver has been systematically investigated as major target organ for ambient chemicals, surprisingly, less information is available in the literature with respect to the adipose tissue.

## Effects of endocrine disruptors, micro- and nano-plastics on mitochondria in the adipose tissue

The two main types of adipose tissue are the white adipose tissue (WAT) and the brown adipose tissue (BAT). While WAT stores excess energy as triglycerides and signals the status of these stores to liver and skeletal muscle via cytokines and hormones, BAT is responsible for energy expenditure and heat generation by non-shivering thermogenesis through the mitochondria [5].

The evidence is mounting that long-term exposure to EDs and MNPs results in a high negative impact on cardiometabolic health [54]. In particular, mitochondrial dysfunction in adipocytes leads to the progression of metabolic disorders, due to the complex impairment of oxidative capacity, lipid metabolism, insulin sensitivity, adipocyte differentiation, and thermogenesis. Among the EDs, bisphenols (BP), polychlorinated biphenyls (PCBs), and phthalates rise the most concerns.

### Bisphenols

Bisphenol A or BPA (2,2-bis(4-hydroxyphenyl) propane) is an industrial chemical used to manufacture polycarbonate plastics and epoxy resins, that acts as an endocrine-disrupting chemical, due to its estrogen-like characteristics. Lately, it has gained extra attention because routine exposure to BPA can lead to oxidative stress, mitochondrial dysfunction, and altered cellular homeostasis. BPA elicits a complex mitochondrial dysfunction-mediated cellular toxicity characterized by mitochondrial membrane potential decrease and impaired bioenergetics, alterations in biogenesis, mitophagy, and mitochondrial DNA [55]. In an obese model of laying hens induced using high-fat diet, it was demonstrated that exposure to BPA (5000 µg/kg BW/day, corresponding to the no observable adverse effect level—NOAEL) induced important oxidative stress, along with the inhibition of antioxidant-related enzymes and increase of lipid peroxidation products [56].

Due to the need of finding a less toxic alternative, researchers have investigated the effect of BPA analogs on mitochondrial function and structure. One of this BPA analogs is bisphenol AF (BPAF), a fluorinated organic compound. However, it has been postulated that BPAF has a higher potential for toxicity due to the fact that the –CF3 groups in BPAF are more electronegative and possibly, more reactive than the –CH3 groups in BPA [57]. Chernis et al. have studied the effect of chronic treatment with BPAF on human adipocytes and discovered that BPAF also causes mitochondrial dysfunction [58]. Adipocytes differentiated with BPAF show restricted respiratory capacity and reduced mitochondrial size and activity. More recently, Wen et al. investigated the effect of bisphenol S (BPS), a very popular BPA substitute, on the risk of obesity. Exposure to BPS at an environmentally relevant dose has been reported to aggravate the diet-induced obesity in female mice in an estrogen-dependent manner. BPS treatment (125 µg/kg/day for 10 weeks) altered BAT morphology and decreased the mitochondrial content and expression of uncoupling protein 1 (UCP-1). In vitro experiments also showed that brown adipocytes treated with BPS exhibited the whitening phenotype, as evidenced by the larger lipid droplets, fewer mitochondria, decreased UCP-1 protein expression, and impaired mitochondrial respiratory function [59]. There are also epidemiological studies reporting the obesogenic effects of bisphenols alone or in association with other EDs [60, 61]

### Polychlorinated biphenyls

Polychlorinated biphenyls (PCBs) are highly toxic and persistent organochlorine chemicals reported to be both carcinogenic and EDs, used in industrial and consumer products, whose manufacturing was banned internationally since 2001. However, PCBs may be present in products and materials fabricated before the PCBs ban, and so can still be released into the environment. Previous studies found a link between exposure to persistent organic pollutants, such as PCBs and the development of insulin resistance and type 2 diabetes [62–64]. Exposure to PCB126 has been earlier associated with mitochondrial dysfunction in rat liver [65] and in rat skeletal muscle tissue [66].

However, since PCBs are very stable compounds that exhibit lipophilic properties they tend to accumulate in high amounts in the adipose tissue [67]. Since PCBs are stored in fat, in an elegant study, Caron et al. hypothesized that PCB126 alters adipokine secretion, which in turn will affect muscle metabolism. To this aim, they acutely exposed (24 h) the 3T3-L1 line of adipocytes to PCB126 (1–100 nM) in two insulin sensitivity conditions: insulin sensitive (IS) and insulin resistant (IR) and measured the secreted adipokines, mitochondrial function, and insulin-stimulated glucose uptake. Communication between adipocytes and muscle cells was mimicked by exposing the C2C12 myotubes to conditioned medium (CM) derived from IS or IR 3T3-L1 adipocytes incubated with PCB126 followed by the assessment of mitochondrial function and insulin-stimulated glucose uptake in myotubes. They showed that IR (but not IS) 3T3-L1 adipocytes treated with PCB126 had significantly higher secretion of IL-6, MCP-1, and TNF-α and lower glucose uptake, and glycolysis. In IR (but not IS) adipocytes, a 24 h PCB126 exposure elicited respiratory dysfunction demonstrated by lower oxygen consumption rate (OCR) and proton leak (state 4) after treatment with 1 or 10 nM PCB126; the impairment of energy metabolism in IR 3T3-L1 adipocytes was linked to the decreased phosphorylation of AMP-activated protein kinase. Finally, the myotubes exposed to the CM from PCB126-treated IR adipocytes had lower glucose uptake, with no alteration of mitochondrial respiration. The authors concluded that PCB126 promoted inflammation and metabolic dysfunction in adipocytes who were already insulin resistant before the acute exposure to the pollutant and that the PCB126-induced inflammation in adipocytes also resulted in impaired glucose uptake and the insulin response in the skeletal muscle [68].

### Phthalates

Phthalates are chemicals commonly used as plasticizers with a negative long-term impact on human health, by acting as endocrine disruptors [69]. Chiang et al. have studied the in vitro effects of mono (2-ethylhexyl)phthalate (MEHP) on energy metabolism of fat cells. They found an increased oxygen consumption rate, and also increased expression of critical genes involved in mitochondrial biogenesis and/or energy metabolism, peroxisome proliferator-activated receptor γ coactivator-1α (PGC-1α), sirtuin 3 (Sirt3), and protein kinase A (PKA) [70]. Hsu et al. have showed in their study that MEHP-treated adipocytes exhibited brown-like characteristics, such as increased mitochondrial leak and higher mitochondrial respiration activity. Moreover, they determined the mRNA levels of genes involved in oxidative phosphorylation, it being the final and most critical step of cellular respiration and found a significant increase [71].

Qi et al. reported that MEHP affected the differentiation 3T3-L1 adipocyte and lead to lipid accumulation through the TYK-2/STAT-3 pathway. Moreover, MEHP was found to decrease the mitochondrial membrane potential, increase intracellular ROS levels, and down-regulate the phosphorylation of STAT-3 in mitochondria [72].

Another toxic compound of very high concern, because of its endocrine disruptive effects, is di-(2-ethylhexyl)-phthalate (DEHP). An in vivo study, conducted on adult male albino rats of Wistar strain, exposed to DEHP (10 or 100 mg/kg body weight for 30 days), from which visceral adipose tissue was retrieved, showed that DEHP treatment increased the hydrogen peroxide and hydroxyl radical levels, while also promoting lipid peroxidation [73]. Additionally, they demonstrated that vitamins C and E prevented the DEHP-induced changes. Schaffert et al. screened 20 plasticizers and their metabolites, including poorly characterized emerging substitutes, in the human Simpson-Golabi-Behmel syndrome (SGBS) cell strain—mature adipocytes and found decreased oxidative phosphorylation and impaired metabolic processes [74].

Tian et al. have recently assessed the effect of MEHP and monocyclohexyl phthalate (MCHP) on glucose and lipid metabolism in adipocytes. While both MEHP and MCHP increased lipid droplet formation, transcriptomic analysis revealed that MEHP predominantly altered fatty acid metabolism pathways, whereas MCHP had less effect [75].

### Tolylfluanid and tributyltin

Tolylfluanid (TF) is a phenylsulfamide fungicide, most frequently used in agriculture, that has been reported to elicit diet-dependent metabolic dysfunction represented by glucose intolerance and increased visceral fat in experimental models of murine obesity [76]. Tributyltin (TBT) is an organometallic compound whose high stability has led to contamination of some aquatic ecosystems from where the residues may reach humans via food consumption [77]. TF promotes differentiation of preadipocytes to adipocytes in the 3T3-L1 cell line [78] and insulin resistance in primary rodent and human adipocytes [79]. Chen et al. have conducted a study aimed at evaluating how several EDs interact with the mitochondrial pyruvate carrier (MPC) [80]. For this study, they isolated mitochondria from mouse brown adipose tissue, which contains high amounts of MPC1 and MPC2, and even though most of the EDs evaluated did not acutely affect pyruvate metabolism, the endocrine disruptors TF and TBT markedly suppressed it. Moreover, they showed that the effect of TF on pyruvate metabolism required MPC2, whereas TBT, the most important pesticide in European freshwaters and marine environments, did not [80].

### Perfluoroalkyl and polyfluoroalkyl substances

Perfluoroalkyl and polyfluoroalkyl substances (PFAS) are fluorinated compounds, widely used in a variety of industrial and consumer applications. However, the main source of contamination is the ingestion of fish and seafood [81]. Perfluorooctanoic acid (PFOA) and perfluorooctane sulfonate (PFOS) are degradation products of PFAS, that resemble fatty acids. Both PFOS and PFOA were reported to reduce body weight by decreasing the major adipose tissue depots (BAT) in mice [82, 83]. Because of this, it was hypothesized that PFOA/PFOAS may directly activate the UCP-1, a mitochondrial protein that mediates heat production in brown adipose tissue [84, 85]. PFOA/PFOS treatment induced increased oxidative capacity and increased UCP1-mediated oxygen consumption. The absence of this effect in heart mitochondria (in mitochondria without UCP-1) is another proof that the presence of UCP-1 is mandatory [85]. However, the research concluded that the body weight-reducing effect of PFOA/PFOS was better explained by a UCP1-dependent decrease in food intake, rather than by elevated thermogenesis [84, 85]. Thus, the possibility that excessive UCP1-activation of BAT by PFOA/PFOS may lead to extreme thermogenesis and extra utilization of food resources, resulting in decreased fitness in mammals—most of which do possess BAT—as well as possible detrimental effects in humans, is under research.

### Heavy metals

Heavy metals are present in our environment and are very popular in the industrial world. While trace amounts of elements such as iron, copper, manganese, and zinc are important in biologic processes, at higher concentrations they have multiple toxic effects. More important, heavy metals such as cadmium, mercury, arsenic, and lead act as EDs and their influence on human health has been a topic of continuous research [86].

#### Cadmium

With respect to cadmium (Cd), it has been reported that smokers have a particularly high risk of exposure from inhaling cigarette smoke, while the non-smokers are exposed mainly through diet, from food that originates in contaminated areas. Cd is particularly dangerous since its half- life is between 15 and 30 years. The main organs that store Cd are the thyroid gland, liver, kidney, lungs, and the skeleton [86]. In a pioneering study performed in the mid 80 s in mitochondria isolated from hamster BAT, Drahota group reported that Cd ions activated the glycerol 3-phosphate dehydrogenase (an enzyme relevant for thermogenesis in BAT) in a very narrow concentration range (1–2 mmol/L), but a strong inhibitory effect happened at higher concentrations (3–4 mmol/L). These authors unveiled that the toxic effect of Cd was due to the modification of the calcium binding sites, since its inhibitory effect could not be reversed by the excess of calcium, the ion normally required for the enzyme activity [87].

#### Zinc

Zinc (Zn) is a less toxic trace element which functions as co-factor for over 300 enzymes and even more proteins, being vital for many physiological processes. It is the endogenous Zn that may contribute to the cytotoxic events, particularly in the brain, where cytotoxicity has been related to the accumulation of free zinc as a consequence of ischemia or trauma, resulting in neuronal cell death [88]. Moreover, recent research has shown that copper (Cu^2+^) greatly amplifies Zn^2+^ neurotoxicity in mouse hypothalamic neuronal cells, indicating that interaction between Zn^2+^ and Cu^2+^ is significant in the progression of neurological diseases. Pyruvic acid significantly suppressed cytochrome c release into the cytoplasm, an index of mitochondrial injury, in a dose-dependent manner, suggesting its potential in preventing Cu^2+^/Zn^2+^-induced neuronal cell death [89]. Additionally, seleno-L-methionine treatment restored the Cu^2+^/Zn^2+^-induced decrease in cellular viability and attenuated the Cu^2+^/Zn^2+^-induced cytotoxicity [90]. However, how these compounds influence the adipose tissue at a mitochondrial level is not well documented.

Low levels of Zn influence several organs, including the endocrine system and especially the thyroid hormones level as well as adaptation to cold [91]. The negative impact of high concentrations of Zn has been less investigated. As such, a pioneering study reported back to 1985 that in vivo administration of Zn inhibited mitochondrial O_2_ consumption in adipose tissue from male Wistar rats [92]. Almost a decade later, Chen et al. investigated the in vitro effect of zinc addition on guanosine diphosphate (GDP) binding to mitochondria in brown adipocytes of genetically obese (ob/ob) mice. They used the mitochondrial GDP binding capacity as a measure of the functional UCP content, in order to evaluate changes in the thermogenic response. Zinc supplementation had no significant influence on GDP binding in lean mice. However, GDP binding decreased as Zn levels increased in ob/ob mice. These authors reported that zinc supplementation significantly reduced brown adipose tissue thermogenesis in ob/ob mice [93].

### Ambient air pollution

Currently, air pollution (a complex mixture of gaseous and particulate components) is a major problem worldwide [94]. Exposure to air pollution is the biggest threat to environmental health, having detrimental effects on the cardiovascular and respiratory systems, but being also related to the development of metabolic diseases such as obesity and type II diabetes mellitus; as such, air pollution may also well be described as an endocrine disruptor [95]. Studies have investigated the changes in the structure and function of BAT and WAT in response to chronic exposure to fine ambient particulate matter in rodent models. Airborne fine particulate matter is smaller than 2.5 μm in aerodynamic diameter (PM2.5). Exposure to PM2.5 increased ROS production [96] and decreased the expression of UCP-1 in brown adipose tissue [96, 97]. The mitochondria number and size were reduced in BAT, while in WAT only the number was significantly reduced [96]. Since it has been reported that PM2.5 toxicity may also depend on its components, including metals such as nickel (Ni), Xu et al. have investigated the effects of Ni, alone or in co-exposure with concentrated ambient air PM2.5 (CAPs). They reported that the influence of Ni exposure was similar to that of CAPs. In addition, Ni exacerbated some (but not all) of the negative effects of CAPs. [98].

The deleterious effects of EDs/MDCs on mitochondria function in adipocytes/adipose tissue are summarized in Table 1.

**Table 1:** Summary of studies regarding the mitochondrial effects of EDs/MDCs in adipocytes/adipose tissue

Endocrine disruptor	Exposure model	Concentration and duration	Experimental Model/Tissue	Relevant findings	Ref
Bisphenol A (BPA)	In vivo	5000 µg/kg BW/day for 14 weeks	Abdominal adipose tissue from an obese model of aged laying hens	- Inhibited activities of T-SOD and GSH-Px - Increased MDA	[56]
Bisphenol AF (BPAF)	In vitro	5 µM for 14 days	Subcutaneous primary human preadipocytes	- Reduced mitochondrial biogenesis - Reduced maximal respiratory capacity	[58]
Bisphenol S (BPS)	In vivo	125 µg/kg/day for 10 weeks	Brown adipose tissue from female mice	- Fewer mitochondria - Decreased UCP-1 protein expression	[59]
	In vitro	15 nM for a total of 7 days	Primary preadipocytes (3T3-L1) from brown adipose tissue of female mice	Cotreatment with E2: - Fewer mitochondria - Decreased UCP-1 protein expression - Suppressed mitochondrial respiratory function	
Polychlorinated biphenyls (PCBs)	In vitro	1–100 nM for 24 h	Murine 3T3-L1 adipocytes in IR and IS conditions	IR 3T3-L1 adipocytes: - Decreased OCR and proton leak (state 4) (1 and 10 nM) - Increased ATP5A expression (10 and 100 nM) - Increased levels of superoxide dismutase 2 (100 nM)	[68]
Mono(2-ethylhexyl) phthalate (MEHP)	In vitro	30–300 µM for 6 days	Murine 3T3-L1 adipocytes	- Increased OCR (100 and 300 µM) - Increased mRNA levels of PGC-1α, Sirt3 and PKA (300 µM)	[70]
	In vitro	50 and 250 µM for 8 days	Murine 3T3-L1 adipocytes	- Decreased protein levels of mitochondrial pSTAT-3 - Increased ROS levels - Decreased mitochondrial membrane potential	[72]
	In vitro	100 µM for 6 days	Murine 3T3-L1 adipocytes	- Increased basal respiration, ATP production, proton leak and non-mitochondrial respiration - Reduced reserve capacity	[71]
Di-(2-ethylhexyl)-phthalate (DEHP)	In vivo	10 and 100 mg/kg body weight for 30 days	Visceral adipose tissue from adult male albino rats of Wistar strain	- Increased H_2_O_2_, hydroxyl radical levels and lipid peroxidation	[73]
Di-(2-ethylhexyl)-phthalate (DEHP) substitutes	In vitro	10 nM and 10 µM for 8 days	Simpson-Golabi-Behmel syndrome (SGBS) cell strain—mature adipocytes	- Increased oxidative stress - Decreased oxidative phosphorylation	[74]
Tolylfluanid (TF)	In vitro	25 µM	Brown adipose tissue of adult female C57/BL6/J mice	- Suppressed pyruvate metabolism - Reduced succinate-mediated respiration	[80]
Tributyltin (TBT)	In vitro	25 µM		- Suppressed pyruvate metabolism	
Perfluorooctane sulfonate (PFOS) and perfluorooctanoic acid (PFOA)	In vivo	0.02% (w/w) for 10 days	The interscapular, periaortic, axillary and cervical brown adipose tissue of mice	- Increased oxidative capacity - Increased UCP1 protein level	[84]
	In vitro	Additions of 80 µM in the concentration range 80–480 µM	The interscapular, periaortic, axillary and cervical brown adipose tissue of mice	- Increased UCP1-mediated oxygen consumption - High concentration of PFOA/PFOS induces production of mitochondrial ROS	[85]
Zinc	In vivo	Zn sulfate dissolved in saline, 10 or 20 mg per kg, given once daily for 2 days	Interscapular brown adipose tissue of male Wistar rats	- Reduced mitochondrial oxygen consumption	[92]
	In vitro	Incubation of mitochondria with 50, 100, or 200 µM zinc sulfate for 4 h	Interscapular brown adipocytes of male mice (obese; lean)	- GDP binding decreased with increasing zinc addition in obese mice	[93]
Cadmium	In vitro	3–4 mmol/L	Brown adipose tissue of hamster	- Strong inhibitory effect on mitochondrial glycerol 3-phosphate dehydrogenase	[87]
Ambient fine particulate matter (PM)	In vivo	Concentrated ambient PM2.5 for 6 h/day, 5 days/week for a total duration of 2 months	Brown and white adipose tissue of male ApoE knockout (ApoE-/-) mice	- Increased production of reactive oxygen species in brown adipose depots - Decreased expression of UCP1 in brown adipose tissue - Significantly reduced mitochondrial number in white and brown adipose tissues - Reduced mitochondrial size in brown adipose tissue	[96]
	In vivo	Concentrated ambient PM2.5) for 6 h/day, 5 days/ week from April 2009 to January 2010	Adipose tissue of male C57BL/6 mice	- Decreased mitochondrial count in visceral adipose - Decreased mitochondrial size in interscapular adipose depots - Reduction of UCP1 expression	[97]
Nickel (Ni) and concentrated ambient air PM2.5 (CAPs)	In vivo	CAPs at a mean of 70 µg/m^3^ + Ni at 0.44 µg/m^3^, 6 h/day, 5 days/week, for 3 months	Brown and white adipose tissue of male ApoE knockout mice	- Significantly reduced UCP1 expression in interscapular BAT and perivascular BAT depots by exposure to the CAPs + Ni - Exposure to either CAPs or Ni alone, or combination of both significantly induced superoxide production in interscapular BAT - NiSO4, with or without CAPs exposure, significantly decreased mitochondrial number in the white adipose depots - CAPs and/or NiSO4 exposure resulted in reduced size of mitochondria in the white adipose depots	[98]

*BAT* brown adipose tissue, *GSH-Px* glutathione peroxidase, *MDA* malondialdehyde, *NiSO4* nickel sulfate, *IR* insulin resistant, *IS* insulin sensitive, *MPC* mitochondrial pyruvate carrier, *OCR* oxygen consumption rate, *P-STAT3* phosphorylated signal transducer and activator of transcription 3, *ROS* reactive oxygen species, *STAT-3* signal transducer and activator of transcription 3, *T-SOD* total superoxide dismutase, *UCP1* uncoupling protein 1

### Micro-and nano-plastics

The infiltration of MPs and NPs into the environment has raised significant concerns regarding their potential health effects. Recently, Marfella et al. highlighted a potential link between the presence of MNPs in blood vessels and cardiovascular disease [14]. They designed a prospective, multicenter, observational study involving patients who were undergoing carotid endarterectomy for asymptomatic carotid artery disease and found that in atherosclerotic plaques surgically removed from the carotid arteries of 304 individuals, plastic particles were evidenced in about half of the cases. Specifically, polyethylene was present in 150 samples and polyvinyl chloride in 31. Electron microscopy revealed that these plaques contained jagged-edged particles identified as MNPs. The presence of plastic in these plaques was significantly associated with a higher risk of developing cardiovascular disease. Over a period of 34 months, individuals with evidence of MNPs in their plaques had a 4.5 times greater risk of experiencing nonfatal myocardial infarction, nonfatal stroke, or death from any cause compared to those without such evidence. However, research on the subcellular or molecular effects of MNPs on the human body remains limited.

A recent investigation was aimed at elucidating the absorption and distribution of MNPs—fluorescent polystyrene (PS) beads in the murine digestive system and adipose tissue. The mice were exposed to 100 nm, 3 μm, and 10 μm PS beads at a dosage of 200 mg/kg via a single gavage and were subjected to in vivo imaging (for the dynamics of the fluorescence intensity) followed by the histological analysis of samples from the stomach, intestines, liver, subcutaneous adipose tissue to corroborate the findings. The authors reported a rapid absorption of 100 nm PS beads, with the highest fluorescence intensity observed in the stomach and small intestine 0.5 h post-exposure. Notably, these nano-plastics were also detected in the liver and adipose tissue within the same timeframe, indicating their swift distribution beyond the digestive tract. At variance, microplastics were predominantly confined to the digestive tract even 4 h after exposure. This study highlights the distinct behavior of nano-plastics in biological systems, emphasizing their potential for rapid accumulation in adipose tissue and subsequent implications for health [99]. A year later, Sen et al. conducted a study aimed at providing evidence on the toxicokinetics of MNPs in mammalian bodies [100]. After confirming fluorescent dye leaching and the impact of pH value, they measured increased levels of fluorescence intensity in the blood, all examined adipose tissues, cerebrum, cerebellum, and testis in the 100 nm group. However, this increase was not observed in the 3 and 10 μm groups, except in the cerebellum and testis at 4 h for the 3 μm PS beads. The presence of PS beads was further corroborated. They found that after a single oral exposure, NPs are quickly absorbed into the bloodstream, accumulate in adipose tissues, and can penetrate both the blood–brain and blood-testis barriers. The study confirmed that the toxicokinetics of MPs in mammals is significantly influenced by particle size. The same year, Shiu et al. demonstrated the distribution of fluorescent polystyrene nano-plastics (60 nm) within the murine WAT and showed that NPs reduced lipolysis under β-adrenergic stimulation [101]. Moreover, chronic oral exposure to NPs of the obese mice at dietary exposure-relevant concentrations impaired fasting-induced lipid mobilization, induced macrophage infiltration in the small intestine, and increased lipid accumulation in the liver, thus exacerbating the metabolic dysfunction.

Few studies have addressed the impact of these compounds on mitochondria within adipose tissue. Basini et al. recently investigated the effects of NPs on primary adipose stromal cells isolated from swine adipose tissue, specifically evaluating cell viability, proliferation, metabolic activity, inflammation mediators, and oxidative stress markers. They reported a decrease in cell viability after prolonged NP exposure, increased TNF-α levels, while PAI-1 was decreased. The redox dysregulation was characterized by increased generation of superoxide, hydrogen peroxide and nitric oxide, lower non-enzymatic antioxidant power, and an increase in catalase activity (a classic scavenger) [102]. In a very recent study, Zhang et al. investigated the effects of chronic administration of PS-NPs on the function of beige adipocytes. In this study, C57BL/6 J male mice were fed a high-fat diet (HFD) with or without PS-NPs exposure for 12 weeks to examine differences in metabolic performance. Additionally, stromal vascular fractions were isolated from C57BL/6 J male mice to differentiate and prepare primary beige adipocyte cultures, which were treated with PS-NPs on the sixth day of differentiation. The results revealed that oral intake of PS-NPs exacerbated metabolic disorders in mice on a HFD, manifesting as suppressed energy expenditure, increased fat mass and liver steatosis, decreased insulin sensitivity, disrupted glucose homeostasis, and reduced cold tolerance compared to the control group. Notably, after 12 weeks of exposure, PS-NPs accumulated in the inguinal fat and decreased the level of UCP-1, a key regulator in the browning process of beige adipocytes. These effects led to decreased energy expenditure and impaired lipid and carbohydrate metabolism. These authors showed for the first time that PS-NPs disrupted mitochondrial function and induced oxidative damage and inflammation in beige adipocytes [103].

The deleterious effects of MNPs in adipocytes/adipose tissue are summarized in Table 2.

**Table 2:** Summary of studies regarding the deleterious effects of MNPs in adipocytes

Exposure model	Concentration	Experimental model/tissue	Relevant findings	Ref
In vitro	5, 25 and 75 μg/μL NPs	Primary adipose stromal cells isolated from swine adipose tissues	Increased TNF-α Inhibited PAI-1 Increased production of O_2_-, H_2_O_2_ and NO Reduced non-enzymatic antioxidant power Increased catalase activity	[102]
In vivo	5 mg/kg nPS	Fat tissue samples and primary beige adipocytes (prepared from stromal vascular fraction) from C57BL/6 J male mice fed with HFD + PS-NPs for 12 weeks	Decreased insulin sensitivity Disrupted glucose homeostasis Suppressed thermogenic genes (mainly decreased level of UCP-1) due to accumulation of PS-NP in the inguinal white adipose tissue	[103]

H_2_O_2_ hydrogen peroxide, *HFD* high-fat diet, *NO* nitric oxide, *NPs* nano-plastics, O_2_- superoxide anion, *PAI-1* plasminogen activator inhibitor 1, *PS-NPs* polystyrene nanoparticles, *TNF-α* tumor necrosis factor α, *UCP-1* uncoupling protein 1

## Future directions

Future research on combating the deleterious effects of environmental toxicants on the adipose tissue should focus on identifying targets and protective strategies at subcellular level. Notably, interventions such as vitamin D, mitochondria-targeted compounds, and more recently, the SGLT2 inhibitors hold promise.

Vitamin D has been shown to exert anti-inflammatory effects [104] and support mitochondrial function [105], thus mitigating oxidative stress and inflammation induced by toxicants. In a recent investigation, Li et al. explored the impact of PS-NPs on lipid metabolism in zebrafish (Danio rerio) and how vitamin D can mitigate these effects [106]. Zebrafish were exposed to PS-NPs (80 nm) at concentrations of 0, 15, or 150 μg/L for 21 days, while being fed either a low (280 IU/kg) or high (2800 IU/kg) vitamin D diet, creating six groups: 0− , 0+ , 15− , 15+ , 150− , and 150+ . Transmission electron microscopy revealed significant PS-NP accumulation in the liver, leading to increased vacuoles and lipid droplets, especially in the 150 − group. A high vitamin D diet significantly reduced these effects, decreasing lipid droplets by 76.92% and triglycerides by 58.52% in the 150 + group compared to the 150 − group. Untargeted lipidomics showed that PS-NPs mainly disrupted lipid molecules related to cell membrane function and biosynthesis, while a high vitamin D diet alleviated these disruptions. The study concludes that PS-NP exposure can cause cell membrane damage and lipid accumulation in zebrafish livers, but high vitamin D levels can mitigate these effects by regulating lipid molecules. These findings highlight the potential of vitamin D to counteract lipid metabolism impairment induced by PS-NPs, providing insights into reducing the toxic effects of nano-plastics on aquatic organisms and suggesting that vitamin D treatment could help alleviate MASLD. Further research is needed to understand and address the environmental risks of PS-NPs.

Mitochondria are vital cellular organelles, and their dysfunction, marked by excessive ROS production, reduced ATP synthesis, and increased cell death, is crucial in the pathogenesis of numerous diseases. Mitochondria-targeted compounds can directly enhance mitochondrial resilience, improve energy metabolism, and reduce apoptosis. Significant therapeutic results have been achieved using lipophilic antioxidant cations, such as MitoQ and SkQ1, as well as the penetrating peptide SS-31 [107]. However, no studies exploring the use of these agents in preventing or mitigating the adverse effects of endocrine disruptors are available in the literature. Further research is necessary to understand the signaling pathways influenced by these compounds, including their long-term impacts on gene expression, proteomics, metabolomics, and epigenetics, particularly concerning endocrine disruption.

Additionally, SGLT2 inhibitors, the novel class of antidiabetics with pleiotropic effects, have unequivocally demonstrated protective properties in alleviating mitochondrial dysfunction, oxidative stress, and inflammation, regardless the presence or the absence of diabetes [108–111]. Exploring these mechanisms further could lead to the development of targeted therapies that protect against environmental toxicant-induced adipose tissue dysfunction, promoting metabolic health and reducing the risk of associated chronic diseases. A recent study by Dhakal et al. explored the role of SGLT2i in alleviating the endothelial senescence and dysfunction induced by NPs [112]. Porcine coronary arteries and isolated endothelial cells were exposed to NPs with and without the SGLT2i enavogliflozin (ENA). The authors reported that NPs significantly increased SGLT2 expression, while ENA markedly reduced NPs-induced senescence-associated-β-gal activity, cell-cycle arrest, and the senescence markers p53 and p21. This suggests that SGLT2 inhibition can prevent NPs-induced endothelial senescence. Additionally, ENA lowered ROS formation by downregulating Nox2 and p22phox and also increased the eNOS expression, thus improving vascular function. The study concludes that NPs-induced premature endothelial senescence is partly mediated by the sodium-glucose cotransporter, indicating that SGLT2 inhibition could be a promising therapeutic approach for preventing and treating cardiovascular disorders aggravated by environmental pollutants.

In the past decades, EDs and MDCs have been reported to contribute to the progression of metabolic diseases via multiple mitochondrial defects [25], including in the WAT [113]. The mechanisms underlying the deleterious effects of the environmental toxicants are summarized in Fig. 1. Future research on persistent pollutants will use the emerging omics technologies (genomics, epigenomics, mitochondriomics and metabolomics) allowing the interpretation of large-scale biological measurements of [114, 115] and provide the opportunity to develop sex-specific strategies to dampen the progression of metabolic diseases [116], including MASLD [117]. Last but not least, view their persistence in multiple organs and tissues, the contribution of EDs and MNPs to the phenomenon of metabolic memory in patients with metabolic diseases [118] remains to be elucidated.

## Conclusions

Morbidity due to the pollution risk factors, which are the unintended consequence of population growth, uncontrolled urbanization, and industrialization, has been reported to be on a constant rise since 2000, particularly in low- and middle-income countries.

Pollutants poisoning water, land, and the ambient air largely coincide with the increasing group of endocrine disruptors. Reducing the use of plastics, improving waste management practices, and promoting alternatives to single-use plastics have been proposed to solve the complex problem of plastic pollution but they will not solve the problem of their persistence. Therefore, extensive research is required in order to elucidate the pathophysiological mechanisms of the combined exposure to endocrine disruptors and micro- and nano-plastics in humans, particularly in the tissues where these compounds are stored and, more important, to identify therapeutic approaches to alleviate these tissues dysfunction.

## Data Availability

No datasets were generated or analysed during the current study.
