# Correction to: Adipose tissue as target of environmental toxicants: focus on mitochondrial dysfunction and oxidative inflammation in metabolic dysfunction-associated steatotic liver disease

**DOI:** 10.1007/s11010-025-05221-2

**Published:** 2025-02-05

**Authors:** Bogdan M. Lolescu, Adina V. Furdui-Linţa, Cosmin A. Ilie, Adrian Sturza, Flavia Zară, Danina M. Muntean, Alexandru Blidişel, Octavian M. Creţu

**Affiliations:** 1 Doctoral School Medicine, Center for Translational Research and Systems Medicine, “Victor Babeș” University of Medicine and Pharmacy of Timişoara, Timişoara, Romania; 2 Center for Translational Research and Systems Medicine, “Victor Babeș” University of Medicine and Pharmacy of Timişoara, Timişoara, Romania; 3 Department III Functional Sciences–Chair of Pathophysiology, “Victor Babeș” University of Medicine and Pharmacy of Timişoara, Timişoara, Romania; 4 Department III Functional Sciences–Chair of Public Health & Sanitary Management, “Victor Babeș” University of Medicine and Pharmacy of Timişoara, Timişoara, Romania; 5 Department II Microscopic Morphology–Chair of Histology, “Victor Babeș” University of Medicine and Pharmacy of Timişoara, Timişoara, Romania; 6 Department of Pathology, Timisoara Municipal Emergency Clinical Hospital, Timişoara, Romania; 7 Department of Surgery I–Clinic of Surgical Semiotics & Thoracic Surgery, Center for Hepato-Biliary and Pancreatic Surgery, “Victor Babeș” University of Medicine and Pharmacy of Timişoara, Eftimie Murgu Sq., No.2, 300041 Timişoara, Romania

**Correction to: Molecular and Cellular Biochemistry** 10.1007/s11010-024-05165-z

In this article the author name Bogdan M. Lolescu was incorrectly written as Bogdan A. Lolescu.

The original has been corrected.

